# Molecular Cytogenetic Analysis of the European Hake *Merluccius merluccius* (Merlucciidae, Gadiformes): U1 and U2 snRNA Gene Clusters Map to the Same Location

**DOI:** 10.1371/journal.pone.0146150

**Published:** 2015-12-30

**Authors:** Daniel García-Souto, Tomás Troncoso, Montse Pérez, Juan José Pasantes

**Affiliations:** 1 Departamento de Bioquímica, Xenética e Inmunoloxía, Universidade de Vigo, Vigo, Spain; 2 Grupo de Acuicultura Marina, Centro Oceanográfico de Vigo, Instituto Español de Oceanografía, Vigo, Spain; Universita degli Studi di Roma La Sapienza, ITALY

## Abstract

The European hake (*Merluccius merluccius*) is a highly valuable and intensely fished species in which a long-term alive stock has been established in captivity for aquaculture purposes. Due to their huge economic importance, genetic studies on hakes were mostly focused on phylogenetic and phylogeographic aspects; however chromosome numbers are still not described for any of the fifteen species in the genus *Merluccius*. In this work we report a chromosome number of 2n = 42 and a karyotype composed of three meta/submetacentric and 18 subtelo/telocentric chromosome pairs. Telomeric sequences appear exclusively at both ends of every single chromosome. Concerning rRNA genes, this species show a single 45S rDNA cluster at an intercalary location on the long arm of subtelocentric chromosome pair 12; the single 5S rDNA cluster is also intercalary to the long arm of chromosome pair 4. While U2 snRNA gene clusters map to a single subcentromeric position on chromosome pair 13, U1 snRNA gene clusters seem to appear on almost all chromosome pairs, but showing bigger clusters on pairs 5, 13, 16, 17 and 19. The brightest signals on pair 13 are coincident with the single U2 snRNA gene cluster signals. Therefore, the use of these probes allows the unequivocal identification of at least 7 of the chromosome pairs that compose the karyotype of *Merluccius merluccius* thus opening the way to integrate molecular genetics and cytological data on the study of the genome of this important species.

## Introduction

The European hake *Merluccius merluccius* (Linnaeus, 1758) is a very valuable commercial groundfish species inhabiting the north-east Atlantic, the Mediterranean and the Black Sea [1]. Together with other hakes of the genus *Merluccius*, this species was overfished until almost reaching exhaustion of their available natural stocks [2], thus leading to an increased interest in hake aquaculture [3–5].

Most genetic studies on hakes were directed to the elucidation of the phylogenetic relationships among hake species using proteins [6] or mitochondrial and/or nuclear DNA markers [7–11]. These studies gave strong evidence of a recent and fast radiation process within the genus *Merluccius* and, additionally, allowed to standardize some of these markers for discriminating species specific hake-derived processed products [12,13], thus securing traceability and increasing the capacity to avoid food fraud. At the same time, the lack of information about the structure and organization of the hake genome reach aspects as basic as chromosome numbers, still not described for any of the fifteen species in the genus *Merluccius* [14].

During the last two decades, molecular cytogenetic techniques have been widely applied to the study of fishes [15–18]. The combination of the information obtained from both classical karyotype analysis and fluorescence *in situ* hybridization (FISH) mapping of different DNA sequences has greatly improved the understanding of evolutionary pathways in some families of fishes [19–24]. Among these sequences, some of the best chromosomal markers are non-coding RNA genes [15–17].

The nuclear genes for ribosomal RNA are organized in two multigene families in eukaryotes [25]. 45S rDNA units consist of three genes expressing for the 18S, 5.8S and 28S rRNAs separated by two transcribed and one intergenic spacer. Many copies of this unit, repeated in tandem, are detected as the nucleolar organizing regions (NORs) at one or various chromosomal positions. 5S rDNAs are also clustered in tandem at one or more chromosomal positions and are composed of a sequence which expresses for the 5S rRNA and a non-transcribed spacer. Although both 45S and 5S rDNA have been located by FISH in many teleosts [15–17] in the order Gadiformes the only available data correspond to the Atlantic cod *Gadus morhua* [26].

The spliceosome is a complex of small nuclear ribonucleoproteins (snRNPs) that controls pre-mRNA splicing; each snRNP is composed by one uridine-rich small nuclear RNA (U snRNA) and associated proteins [27,28]. The genomic organization of the snRNA genes (snDNA), the genes expressing the U snRNAs, shows considerable variation in eukaryote genomes [29]. Molecular analysis of U1 and U2 snRNA genes in fishes indicate that, at least in a few species, some of the copies of these genes are linked [19,29–31] but no FISH mapping evidence has corroborated so far that linkage. U1 snRNA genes have been mapped by FISH to a single location on the chromosomes of 19 species of cichlid fishes [19] and to three chromosome pairs in five species of *Astyanax* [24]. On the other hand, U2 snRNA genes cluster at one or more chromosomal locations in 24 species of teleosts belonging to the families Batrachoidiae [32], Gymnotidae [33], Moronidae [34], Spardidae [21], Scianidae [31], Bagridae [35], Haemulidae [36] and Characidae [24].

Taking into account the absence of karyological data of the European hake [14], in this work we report its chromosome number and establish its karyotype after studying its chromosomes by means of 4’,6-diamidino-2-phenylindole (DAPI) / propidium iodide (PI) and chromomycin A3 (CMA) / DAPI fluorescence staining and FISH using 28S rDNA, 5S rDNA, U1 snDNA, U2 snDNA and telomeric sequences.

## Materials and Methods

### Biological Material

European hake larvae were obtained from hatched eggs obtained from spontaneous spawning of the hake brood stock acclimated at the Instituto Español de Oceanografía in Vigo (NW Spain). Adult specimens were collected at the outer part of Rías de Vigo and Pontevedra (NW Spain) by the authorized artisanal fishing boat “Yamevés” and during the multidisciplinary Spanish acoustic survey PELACUS0314 of the Instituto Español de Oceanografía in the Cantabric Sea (N Spain). The experimental procedure was performed with the approval of the Ethics Committee of the University of Vigo, complying with the current laws of Spain. All institutional and national guidelines for the care and use of laboratory animals were followed.

### DNA Extraction, PCR Amplification and Probe Labelling

Total DNA was extracted following the FENOSALT method [37]. FISH probes were obtained by polymerase chain reaction (PCR) as previously published [38]. U1 and U2 snDNAs were amplified using primers ColU1F/ColU1R [39] and U2F/U2R [19], respectively. In order to assess the existence of linked U1 and U2 snDNA units, different combinations of those primers (ColU1F/U2R; ColU1R/U2F; ColU1F/U2F; ColU1R/U2F) were also used in PCR reactions. Universal primers retrieved from the Vilgalys Lab website (R. Vilgalys, Duke University, Durham, NC [http://www.biology.duke.edu/fungi/mycolab/primers.htm]) were used to amplify a fragment of the 28S rRNA gene of the 45S rDNA repeat. The amplification of the 5S rDNA was performed using primers described in [40].

28S rDNA probes were labelled with biotin-16-dUTP (Roche Applied Science) and/or digoxigenin-11-dUTP (10x DIG Labeling Mix, Roche Applied Science) using a nick translation kit (Roche Applied Science). 5S rDNA, U1 snDNA and U2 snDNA probes were directly labelled by PCR either with biotin-16-dUTP (20 μM) or digoxigenin-11-dUTP (5 μM). The labelled PCR products were precipitated before FISH.

### Chromosome Preparation and Fluorescent *In Situ* Hybridization (FISH)

Larvae were housed in 0.5 L beakers and exposed to colchicine (0.005%) for 6 hT T, immersed in 50% and 25% seawater for 1 h and fixed with ethanol/acetic acid for 1 h. Adults specimens were dissected *perimorten*, sexed and the whole branchial arches were immersed in two consecutive baths of colchicine (0.05%) in 50% and 25% seawater for 2 h 30 min each before fixation with ethanol/acetic acid for 1 h. Chromosome spreads were obtained by dissociating small pieces of tissue in 60% acetic acid and dropping the cellular suspension onto clean slides heated to 50°C. Some of the chromosome preparations were sequentially stained with CMA/DAPI and PI/DAPI as described by [41].

Single and double FISH experiments were performed following published methods [41]. Before FISH, chromosome preparations were stained with DAPI and PI and selected metaphase plates photographed. After washing in 4xSSC/Tween20 and distilled water followed by dehydration in a ethanol series, chromosome preparations were digested with RNase (100 μg/mL, 1 h, 37°C), treated with pepsin (0.05%, 10 min, 37°C) and fixed in formaldehyde (1%, 10 min, 25°C). Preparations were then denatured in 70% (v/v) formamide/2xSSC (69°C, 2 min), dehydrated in a cold ethanol series, air dried and hybridized overnight at 37°C. Post-hybridization washing was carried out in 50% (v/v) formamide/2xSSC (45°C, 3 x 5 min, shaking) and 0.5xSSC (45°C, 3 x 5 min, shaking). Signal detection was performed using fluorescein avidin and biotinylated anti-avidin for the biotinylated probes and mouse antidigoxigenin, goat anti-mouse rhodamine and rabbit anti-goat rhodamine for the digoxigenin-labelled probes. Slides were counterstained with DAPI and mounted in antifade (Vectashield, Vector). In order to map four probes on the same plates, two sequential FISH experiments were performed. The probes employed in the first hybridization were biotin-labelled U1 snDNAs and digoxigenin-labelled U2 snDNAs. After visualization and photography, the preparations were re-hybridized using digoxigenin-labelled 5S rDNA probes and biotin-labelled 28S rDNA probes and the same metaphase plates were photographed again. Telomeric sequences were also mapped by FISH using a telomeric (CCCTAA)_3_ peptide nucleic acid (PNA) probe (Applied Biosystems) following the protocol indicated by the supplier.

Slide visualization and photography were carried out using a Nikon Eclipse-800 microscope equipped with an epifluorescence system. Chromosome counting and karyotype analysis were performed in 40 specimens, 20 larvae and 20 adults (10 males, 10 females). A minimum of 10 individuals and 10 complete metaphase plates per individual were recorded for each probe or combination of probes. Separated images for each fluorochrome were obtained using a DS-Qi1Mc CCD camera (Nikon) controlled by the NIS-Elements software (Nikon). The merging of the images was done with Adobe Photoshop. To establish the karyotype of the European hake, 10 high quality complete metaphase plates showing FISH signals were used to construct karyotypes. Chromosome and arm lengths were carefully measured with Micromeasure 3.3 [42] and relative lengths and centromeric indices were calculated. Chromosome nomenclature follows [43].

### Sequence Analysis

U1 and U2 snDNA PCR amplification products were gel-purified using a FavorPrepTM GEL/PCR Purification Kit (Favorgen) and directly sequenced using an Applied Biosystems TM 3130 Genetic Analyzer with a BigDye Terminator v3.1 Cycle Sequencing Kit (Applied Biosystems). DNA sequences were edited and revised with BioEdit 7.0.0 [44], aligned with MEGA 5.05 [45] and annotated using the Basic Local Alignment Search Tool algorithm (BLAST) [46], available at the National Center for Biotechnology Information (NCBI) (http://www.ncbi.nlm.nih.gov/blast).

## Results

**Fig 1:**
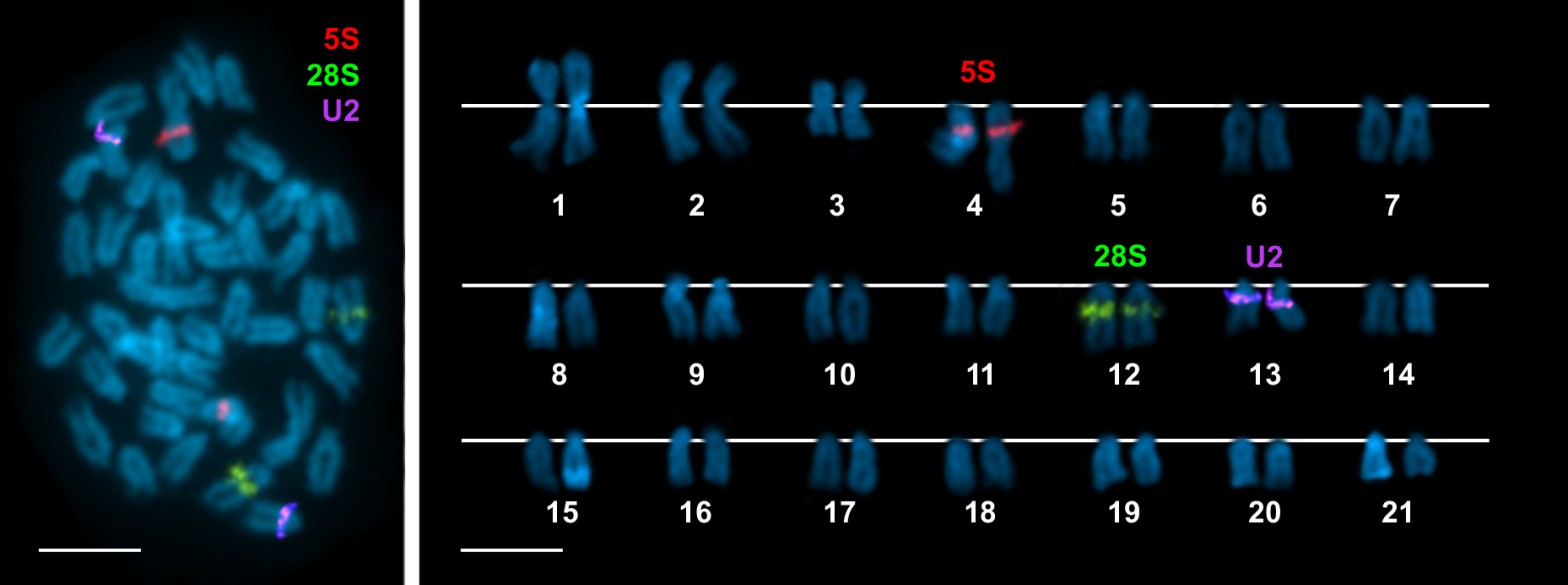
Chromosomal mapping of rRNA and U2 snRNA genes to chromosomes of *Merluccius merluccius*. Double-FISH experiments using a 28S rDNA probe (green) and a 5S rDNA probe (red) demonstrate the presence of a single clusters for both 45S and 5S rRNA genes on different chromosome pairs (**a**). Rehybridization of the same metaphases with an U2 snDNA probe (violet) also give signals at a single location on a different chromosome pair (**a**). The corresponding karyotype shows these signals on chromosome pairs 12, 4 and 13, respectively (**b**). Chromosomes are counterstained with DAPI. Scale bars, 5 μm.

**Fig 2:**
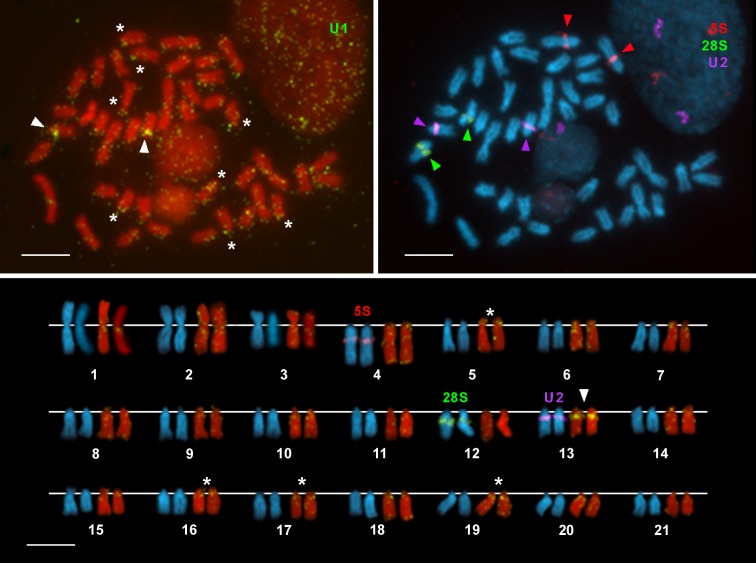
Chromosomal mapping of U1 snRNA genes to chromosomes of *Merluccius merluccius*. FISH experiments using a U1 snDNA probe (green) on chromosomes counterstained with PI (**a**) show signals on many chromosome pairs. The brightest signals appear on one pair (arrowheads in **a**) but there are also strong, consistent, signals (asterisks in **a**) on four more pairs. The corresponding karyotype shows that those signals are on chromosome pairs 13 and 5, 16, 17 and 19. Other signals are also clearly visible in many other pairs (**a**, **c**) but they are fainter and/or not always present in both homologues of each pair. FISH experiment on the same metaphase counterstained with DAPI (**b**, **c**) shows that 28S rDNA (green) and 5S rDNA (red) clusters are separated from the U1 snRNA gene clusters but that the single U2 snRNA gene cluster on chromosome 13 is coincident with the biggest U1 snRNA cluster. Scale bars, 5 μm.

A diploid chromosome number of 2*n* = 42 was determined for the European hake *Merluccius merluccius* after analyzing 400 metaphase plates belonging to 20 larvae and 20 adults (10 females and 10 males) (Figs 1 and 2). The karyotype is composed by three meta/submetacentric and 18 subtelo/telocentric chromosome pairs. No differences were detected among karyotypes from males and females neither from larvae and adults nor from individuals collected at different places.

The combined DAPI/PI staining revealed the presence of DAPI negative region intercalary to the long arm of telocentric chromosome pair 12. CMA staining of the same metaphases allowed detecting the presence of CMA positive bands coincident with the DAPI negative regions (not shown). FISH experiments using 28S rDNA probes demonstrated that the major ribosomal gene cluster is coincident with the DAPI-/CMA+ band on the long arm of telocentric chromosome pair 12 (Fig 1).

FISH mapping of 5S rRNA genes was performed using the whole 5S rDNA repeat as probe. Hybridization signals were studied in 138 complete metaphase plates, at least 10 per individual, obtained from 10 specimens. As shown in Fig 1, *M*. *merluccius* presents a single cluster of 5S rDNA repeats located at a intercalary position of the long arm of telocentric chromosome pair 4.

Double FISH experiments using 5S and 28S rDNA probes labelled differently confirmed the relative positions of the two rRNA gene families on the chromosomes of *M*. *merluccius*. As shown in Fig 1, the chromosome pairs bearing 5S rDNA clusters are different from those carrying major rDNA signals.

U2 snRNA gene signals also appear at a single location, subcentromeric on the long arm of chromosome 13. Rehybridization experiments using U2 snRNA gene probes on slides previously hybridized with 5S and 28S rDNA probes confirmed that these three types of sequences are located on different chromosome pairs (Fig 1).

Hybridization signals corresponding to the U1 snRNA gene probes are scattered throughout most chromosome pairs in *M*. *merluccius* (Fig 2). The brightest signals are subcentromeric on the long arms of chromosome pair 13, being signals on pairs 5, 16, 17 and 19 also strong and present in the two members of each pair in all metaphases. The signals at other chromosomal loci are still consistent from metaphase to metaphase but they are fainter and/or not always present in the two homologues of each pair. Some of the chromosome preparation hybridized with U1 snDNA probes were rehybridized with 28S and 5S rDNA and U2 snDNA probes (Fig 2) allowing to confirm the location of the U1 snRNA gene clusters in relation to the other ncRNA genes analyzed. These experiments clearly confirm, on the one hand, that the single U2 snDNA cluster and the biggest U1 snDNA cluster co-localize on chromosome pair 13 and, on the other, that the 5S rDNA bearing chromosome pair number 4 also shows faint U1 snDNA signals subcentromeric, intercalary and subtelomeric on its long arms.

**Fig 3:**
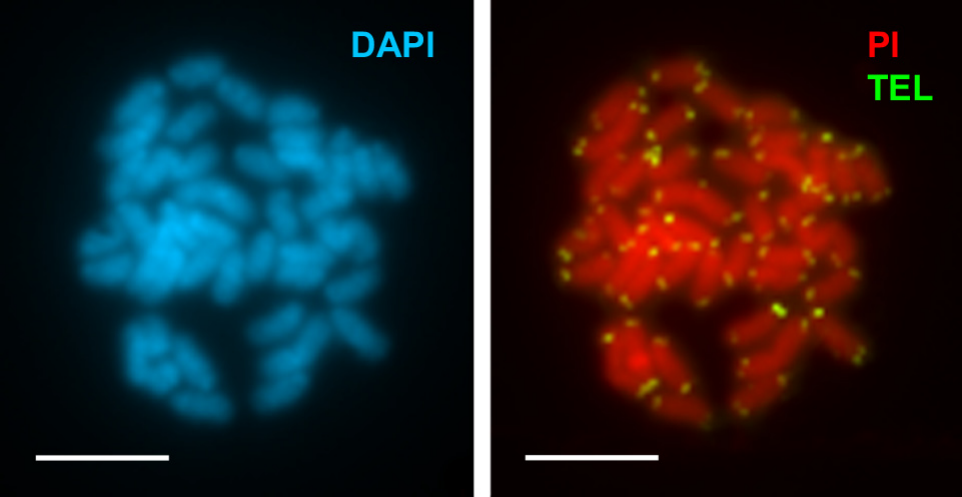
Chromosomal mapping of telomeric sequences to chromosomes of *Merluccius merluccius*. Metaphase plate of *Merluccius merluccius* stained with DAPI (**a**) and PI (**b**). Note that telomeric signals (green) appear only at the ends of the chromosomes. Scale bars, 5 μm.

Telomeric sequences were detected using a vertebrate telomeric (CCCTAA)_3_ PNA probe. Single distinct terminal signals appear at the ends of both sister chromatids of every mitotic chromosome (Fig 3). No additional interstitial telomeric sequences were observed.

After using the internal specific primers ColU1F/ColU1R [39] and U2F/U2R [19] to amplify U1 and U2 snDNAs, two amplicons of 143 (KT873857) and 176 bp (KT873858) were obtained. These sequences show high homology to U1and U2 snDNA sequences available on the NCBI database. The additional 1149 bp U2F/U2R and 1211 bp U2F/colU1R fragments were also sequenced. The U2F/U2R 1149 bp PCR product (KT873855) includes two consecutive U2 snRNA genes separated by a 786 bp fragment containing a tRNA^Asp^ pseudogene (332 to 403) and a complete U5 snRNA gene (608 to 723). The 1211 bp U2F/colU1R amplicon (KT873856) comprises U2 and U1 snDNAs at both ends and shares a 94% similarity with the first 358 bp of the previous sequence. A blast search on the remaining spacer revealed incomplete copies of U6 (652 to 686, antisense) and a degenerated U2 (814 to 847) snDNA, respectively.

## Discussion

This is the first karyological report about a species of the family Merluciidae. No chromosomal numbers are available for any of the other 14 species belonging to this family and the chromosomal characterization of the Gadiformes is limited to the knowledge of mitotic chromosome numbers and karyotypes in only 16 species [14,26]. The diploid chromosome number of 2n = 42 described in this work is into the range (26 to 48) published for other species of Gadiformes. The karyotype of *Merluccius merluccius* is composed of three meta/submetacentric and 19 subtelo/telocentric chromosome pairs without any indication of heteromorphic sex chromosome pairs. The other species of Gadiformes previously studied also show karyotypes composed of both types of chromosomes but in all of them the number of meta/submetacentric chromosome pairs is higher [14,26].

Concerning telomeric sequences, the detection of the vertebrate (TTAGGG)_n_ repeat at chromosome ends in *M*. *merluccius* and their absence at intercalary locations is coincident with results obtained in the majority of species of fishes analyzed [18,23,33,47,48].

Chromosomal mapping of ribosomal RNA genes has been performed in many species of fishes [15–17,20–22,24,26,31–36,49–54]. The presence of signals at a single location in one chromosome pair for the 45S rDNA in *M*. *merluccius* is concordant with results obtained in more than 70% of the species of teleosts analyzed [17,21,23,31,35,53,54]. On the contrary, the intercalary position of the cluster found in the European hake is rare among teleosts, 87% of which show subterminal NORs [17]. The only other species of Gadiformes in which 45S rDNA clusters have been mapped to chromosomes is the Atlantic cod *Gadus morhua* [26]. In contrast to the European hake, the Atlantic cod shows polymorphic 45S rDNA signals on the short arms of two to three chromosome pairs.

Regarding the 5S rDNA [15,16,20–22,24,26,31–36,49,50,52–54], while some fish species show signals restricted to one chromosome pair, in others the signals appear in many or almost all chromosome pairs [i.e. 49,53,54]. The Atlantic cod, the only other gadoid studied to date, show signals at subterminal regions on the short arms of six pairs of chromosomes [26]. The occurrence of a single 5S rDNA cluster at an intercalary location in *M*. *merluccius* is coincident with the presumably ancestral situation in teleosts. This hypothesis is based, on the one hand, in the existence of many species showing a single 5S rDNA locus, including most of the species presenting the 2n = 48 basal karyotype, and, on the other, in the interstitial location of these sequences in most fishes [16].

Conversely, molecular analysis of 5S rDNA sequences demonstrated that the presence of more than one type of 5S rDNA repeats is also a common feature in the fish genome [9,11,15,22,32,36,50,55]. FISH mapping of these 5S rDNA variant sequences also demonstrated that, at least in some cases, the copies of each of these variants constitute independent clusters located on different chromosome pairs [15,50,55]. In any case, this is not a conserved situation in teleosts because species belonging to a single taxon may present one single type of rDNA while others present two or more types [32,52]. In this sense, molecular studies in the genus *Merluccius* [9,11] demonstrated that while some of the species present two different types of 5S expressing sequences [9], and that the whole repeat sequence is very heterogeneous among them [11], other species of the genus, including *M*. *merluccius*, show a single type of 5S rDNA.

The molecular analysis of U1 and U2 snRNA genes in fishes indicate the existence of linked copies of these genes [19,29–31]. The detection of amplicons containing U1 and U2 snDNAs demonstrates that this is also the case in the European hake. Furthermore, the mapping results presented in this work constitute the first case in which the repeats of such linked units appear in enough number to be located by FISH. On the other hand, the presence of amplicons containing U1 and/or U2 snRNA genes linked to complete or incomplete copies of other U snDNAs is also coincident with previous findings in other fish species [19,29–31].

The presence of a single cluster of U2 snRNA genes in the European hake is concordant with the situation in 16 of the other 23 species of teleosts in which these sequences have been mapped [21,24,31–36]. Another species show two U2 snDNA clusters [24] and the remaining 6 species show signals scattered along many chromosomes but in some cases also present a main cluster. All of these species belong to taxa in which other species present single signals [32,33,36].

U1 snDNA has only been mapped to chromosomes of 19 species of cichlid fishes [19]. Although all species show a single U1 snRNA gene cluster, molecular analysis of these sequences in *Oreochromis niloticus* detected the presence of multiple additional U1 snRNA gene and pseudogene clustering that were not detectable by FISH. In contrast, 5 species of *Astyanax* [24] show U1 snRNA gene clusters in three chromosome pairs. The presence of a higher number of U1 snRNA gene signals in *M*. *merluccius* could thus indicate a higher clustering level of these sequences at multiple loci as result of multiple transposition events between non-homologous chromosomes, as proposed for other organisms [56]. In fact, these genes have been suggested behaving like mobile elements in metazoans [29], although there is some controversy regarding them having or not intrinsic transposable capability [19].

The application of next generation sequencing (NGS) methods to many species has increased our knowledge of the genome in many taxa, including fishes [57]. However, the number of genomes sequenced by NGS is already higher than the number of genomes with physical or genetic maps for anchoring the assemblies to chromosomes thus making necessary to develop high-resolution chromosome-based physical maps as an essential framework for the annotation and evolutionary analysis of genomes [58]. In this sense, the results obtained in this work showing the chromosomal location of rDNAs and U snDNA gene families in the European hake are the first step on the characterization of the genome of Merlucciidae.
